# The Contagiousness of Chronic Urethral Discharges1Read before the Section on Genito-Urinary Surgery, New York Academy of Medicine, Thursday evening, Feb. 12, 1891.

**Published:** 1891-06

**Authors:** George Emerson Brewer

**Affiliations:** New York


### THE CONTAGIOUSNESS OF CHRONIC URETHRAL DISCHARGES.[1]

By GEORGE EMERSON BREWER, M. D., New York.

The practising physician is frequently called upon to answer inquiries regarding the limit of the contagious stage of a gonorrheal urethritis.

The question is a serious one, especially when considered in its relation to marriage, and should, I believe, be regarded as equal in importance to that of syphilis.

It has not infrequently been my experience to be consulted by young men, a few weeks or months before a contemplated marriage, with a history of one or more attacks of gonorrhea in former years, and who believe themselves to be well; yet who, upon a careful examination, present the unmistakable signs of a chronic urethritis.

The only evidence of disease remaining in these cases may be, and frequently is, the presence in the urine of small thread-like bodies, to which the name of *tripper faden* has been given by the German surgeons who first described them and demonstrated their importance. These minute shreds are composed of mucus, pus, and epithelium; and represent the secretions which adhere to any granular patch or area of chronic inflammation remaining on the urethral mucous membrane.

As these cases have often caused me no small measure of anxiety, it is the object of this communication to call attention to the opinions of some of our most prominent authorities upon the subject, and to elicit the views of those present whose experience has been greater than my own.

In consulting the standard authors upon the subject of genito-urinary diseases, one is impressed by the marked difference of opinion, regarding the contagiousness of chronic urethral discharges, held by those whose reputation and experience entitle them to the foremost position in the discussion of this question.

As early as 1785, Kühn called attention to the fact that the discharges resulting from a gonorrhea remained contagious so long as they contained pus. The opposite opinion was held by Hunter, who

1. Read before the Section on Genito-Urinary Surgery, New York Academy of Medicine, Thursday evening, Feb. 12, 1891.

denied the possibility of contagion from the gleety discharges of a chronic urethritis. This view was also shared by Bell and Ricord. In a paper entitled Gonorrhea a Non-specific Disease, published in the New York *Medical Independent* in 1864, A. K. Gardiner strenuously denies the contagious element in any save the discharges from the most acute stage of the disease; and says regarding gleet that it is "allowably benign and innocuous."

Noeggerath, on the other hand, in a paper published in 1872,[1] concludes that a man who has once been the subject of a gonorrheal urethritis, *never* fully recovers; that the disease invariably lingers in the glands and ducts emptying into the canal, and may at any time furnish a secretion which may infect those with whom he has sexual relations. He also states that nine-tenths of all women married to men who have had gonorrhea, sooner or later become the subject of incurable and painful inflammatory disease of the uterus, tubes, or ovaries; that this infection may take place rapidly, and manifest itself as an acute affection, or by means of a slow and unrecognized process to which he gives the name of "latent gonorrhea." In a subsequent paper, read before the American Gynecological Association in 1876, the author reiterates these opinions, and adds that 90 per cent. of all cases of sterility can be directly traced to gonorrhea.

Without entering into any discussion regarding the correctness of these views, which have been the object of considerable criticism, the fact remains that these papers had the effect of calling attention to a scource of female disease and suffering, the importance of which had not, until their publication, been adequately recognized.

This difference of opinion upon so important a question is hardly to be wondered at, when we consider that at the time these views were enunciated, nothing definite was known regarding the etiology of this disease.

Since the discovery by Neisser, in 1878, of the gonococcus, and the establishment of its relationship to this disease, but one opinion can logically be held by those who accept his theory of gonorrheal inflammations, and that is, that all secretions containing this microörganism are capable of transmitting the disease under favorable conditions. In his recently published work[2] upon this subject, Ernest Finger emphasizes this point and states, regarding marriage,

1. "Die Latente Gonorrhöe im Weiblichen Geschlecht," Bonn, 1872.
2. "Die Blennorrhöe der Sexualorgane und ihre Complicationen," 1888.

that it should be absolutely prohibited in all cases where the existence of a chronic urethritis is evidenced by the presence of the "morning drop" or *tripper faden* in the urine, until the following facts have been established :

1st. That after from two to four weeks of daily observation, the secretions from the urethra are found to be *free from pus and made up wholly of epithelial cells.*

2d. That no gonococci can be detected by the microscope, even after a purulent discharge has been established by the employment of irritating injections of corrosive sublimate or nitrate of silver ; and

3d. That neither postatitis nor stricture exists.

I must confess that upon first becoming acquainted with the views expressed by this author, I regarded his conditions unnecessarily severe, and his opinion of the danger greatly exaggerated. A somewhat remarkable case, however, soon came under my observation, which illustrated in a most striking manner the fact that unless some of the precautions advised by Finger had been insisted upon, the responsibility of a terrible and dangerous illness would justly have rested upon my head.

Mr. H., aged 30, called on me in October, 1889, and stated that he expected to be married in six weeks, and that although he knew himself to be perfectly well, he desired the confirmatory assurance of a physician after a thorough examination. He told me that, six years ago, he had experienced an attack of urethritis which was unusually acute and severe, and which continued uninterruptedly for a period of more than twelve months. After nearly a year of unsuccessful treatment, he submitted to a circumcision and meatotomy in the hope that the improved condition of the parts might favor a more rapid recovery. At the end of a very tedious course of treatment the discharge ceased, and with the exception of one or two slight exacerbations after alcoholic or sexual excesses, he has since remained free from the evidences of an active urethritis. For the past three years, he alleged, there had been absolutely no urethral discharge save at times a slight moisture at the meatus in the morning.

Upon examination no secretion could be pressed from the urethra. Exploration by means of the urethrameter revealed a normal caliber of 36 F.—meatus 28—a stricture measuring 30 at 3 inches. Endoscopic examination showed granular patches and congested areas in the neighborhood of the bulb and behind the stricture.

An examination of the urine revealed, in the first portion passed, a number of *tripper faden.* One of these was selected, spread out upon a slide, stained, examined microscopically, and found to contain epithelial cells, pus, and several very characteristic colonies of gonococci.

At the conclusion of the examination he was told that it would be impossible for him to think of being married at the time stated, or at any subsequent date, until he had been relieved of his chronic urethritis, and until examination of the urethral secretions failed to demonstrate the presence of gonococci. Upon the receipt of this advice he became somewhat indignant, affirmed that "he knew better," and that repeated experience had taught him that there was nothing contagious remaining in his urethra. I immediately called upon Dr. Holbrook Curtis who was in an adjoining room, to confirm the microscopic diagnosis and bear witness to what was said, and I again repeated in his presence my former statement and added that as his medical adviser I absolutely forbade the marriage, and that if he declined to accept my advice, he must assume the entire responsibility.

Without dwelling upon the details of several interviews which followed, suffice it to say that he reluctantly promised to postpone the ceremony and undergo a proper course of treatment.

The strictures were divided and the subsequent treatment consisted in the passage of full-sized sounds, urethral irrigation, and local applications by means of the endoscope. He slowly but steadily improved, and at the end of five weeks the *tripper faden* had markedly decreased in numbers; gonococci could, however, occasionally be found. At no time was there any discharge from the urethra.

At this period he suddenly announced that it was his intention to carry out his original plan and be married in a week—which he did against my advice and assuming all responsibility.

The sequel to this narrative is peculiarly sad and instructive.

Two weeks after the ceremony I was called to attend his young wife, who presented all the symptoms of a severe gonorrheal infection. Examination revealed the presence of an angry and painfully acute purulent urethritis and vulvitis, pus from the urethra showing abundant colonies of gonococci. A severe cystitis followed, a large vulvo-vaginal abscess, which was opened under ether, also a marked pyelitis, which continued for many months. The patient was in bed under the constant watchfulness of a trained nurse for seven weeks, and during the year which followed was more or less of an invalid, suffering from recurrent attacks of cystitis, and all directly traceable to the unfortunately premature marriage.

I am aware that extraordinary and seemingly remarkable cases of this kind frequently admit of a more simple and rational explanation when all the facts are accessible, and that the discovery of another and more recent source of infection often dispels illusions and destroys theories which have been reared upon the insufficient evidence of a few imperfectly investigated cases. In this case, however, I was in a position to become familiar with all the circumstances, and having a professional aquaintance with both patients and their families for several years, I can state without the slightest hesitation that the possibility of an infection, in a manner other than I have indicated, is wholly out of the question.

In reviewing the records of nearly one thousand cases of urethritis, treated by me during the past five years, I find that in six instances I was consulted regarding the propriety of marriage, under circumstances similar to those which existed in the case reported above. My rule had always been in such cases never to allow marriage until at least three months had elapsed since the cessation of all acute symptoms, and until repeated examinations of the secretions (including the *tripper faden*) had failed to show the presence of gonococci. In the six cases referred to, these conditions were observed—and in no instance has the wife exhibited the slightest evidence of infection.

It is to me somewhat surprising that, in most of the recent works on this disease, little or no attention is given to this very important subject, whereas the questions of syphilis and marriage has occupied the attention of a very large number of writers. I think I am justified in saying that it is the opinion of those acquainted with the subject, that far more suffering and incurable disease in women can be attributed to gonorrheal than to syphilitic infection.

In conclusion, allow me to urge upon all interested in this subject the necessity of unusual care in examinations undertaken with a view to forming an opinion regarding the propriety of marriage, in those who have been the subject of gonorrheal urethritis. The safest method would be to follow the advice of Finger as quoted above; certainly none should assume the responsibility of sanctioning a marriage without at least imposing the conditions which it has been my custom to insist upon, before I became acquainted with the views of this distinguished authority.—*Journal of Cutaneous and Genito-Urinary Diseases.*

